# Case report: Necrotizing leukomyelitis and meningitis in a Pomeranian

**DOI:** 10.3389/fvets.2024.1303084

**Published:** 2024-03-12

**Authors:** Koen M. Santifort, Laurent Garosi, Erik A. W. S. Weerts

**Affiliations:** ^1^IVC Evidensia Small Animal Referral Hospital Arnhem, Neurology, Arnhem, Netherlands; ^2^IVC Evidensia Small Animal Referral Hospital Hart van Brabant, Neurology, Waalwijk, Netherlands; ^3^Vet Oracle Teleradiology, Norfolk, United Kingdom; ^4^Division of Pathology, Department of Biomedical Health Sciences, Faculty of Veterinary Medicine, Utrecht University, Utrecht, Netherlands

**Keywords:** meningomyelitis, MUO, immune-mediated, small breed, dog, lymphohistiocytic

## Abstract

A 2.5-year-old female entire Pomeranian dog was presented for acute paraparesis progressing within 2 days to paraplegia. General physical examination was unremarkable. Neurological examination showed paraplegia without nociception, a mass reflex upon testing perineal reflexes and withdrawal reflexes in the pelvic limbs and patellar hyperreflexia. Cutaneous trunci reflexes were absent caudal to the level of the 6th thoracic vertebra. Spinal hyperesthesia was present. Neuroanatomical localization was consistent with a T3-L3 myelopathy. Hematological and biochemical blood tests [including C-reactive protein (CRP)] were within reference ranges. MRI of the spinal cord from the level of the 1st thoracic vertebra to the sacrum revealed a patchy, ill-defined, moderate to marked T2W hyperintense, contrast enhancing intramedullary lesion extending from T1 to L4. Medical treatment based on a working diagnosis of meningomyelitis of unknown cause was initiated with corticosteroids and methadone based on pain scores. Prognosis was grave and after 3 days without return of nociception, the dog was euthanized according to the owners’ wishes. Post-mortem histopathological examination of the brain and spinal cord yielded a morphological diagnosis of severe, segmental, bilateral and fairly symmetrical, necrotizing lymphohistiocytic leukomyelitis, with a non-suppurative angiocentric leptomeningitis. Some minor, focal, lymphocytic perivascular cuffing was found in the medulla oblongata as well, but otherwise there were no signs of brain involvement. No infectious causes were identified with ancillary tests. This case report underlines the importance of including meningomyelitis in the differential diagnosis list of dogs presented for acute progressive neurological signs referable to a myelopathy.

## Introduction

Acute-onset canine myelopathies are fairly common in veterinary clinical neurology. The thoracolumbar spinal cord is most often affected, resulting in clinical presentations that vary from spinal hyperesthesia, to paresis, to paralysis with or without nociception and incontinence. The most common cause for acute-onset thoracolumbar myelopathy is intervertebral disc disease (IVDD) (1–5). Intervertebral disc extrusion (IVDE) of degenerated nucleus pulposus represents the largest subgroup of IVDD, but other subgroups such as acute non-compressive nucleus pulposus extrusion (ANNPE) and hydrated nucleus pulposus extrusion (HNPE), with their own clinical and diagnostic imaging characteristics are differential diagnoses to consider (2).

Aside from IVDD, various other etiological categories with representative diseases included therein should be included in the differential diagnosis list of patients with acute-onset thoracolumbar myelopathies (1). These include trauma (e.g., vertebral fracture/luxation and resultant compression, tearing, shearing, laceration, and hemorrhage of/in the spinal cord), inflammatory disorders (e.g., discospondylitis and empyema, meningomyelitis infectious versus immune-mediated), toxicity or nutritional deficiency (rarely identified clinically in dogs, more common in farm animals), vascular disorders [hematomyelia, ischemic myelopathy (fibrocartilaginous embolic myelopathy, FCEM)], anomalies with acute complications (hamartoma, cavernous angioma), and (para)neoplastic disorders (e.g., multiple myeloma, lymphoma, primary spinal cord tumors). Each of these disorders could have primary or secondary necrotizing and inflammatory components that can be identified definitively by histopathological examination. Ascending-descending myelomalacia caused by a primary spinal cord insult due to IVDE is an example of severe, usually fatal, and often hemorrhagic, progressive necrosis of the spinal cord parenchyma (6–8).

To clinically differentiate between the different etiological categories, clinical reasoning as well as results of diagnostic tests are combined to yield the most likely or confirmed *in vivo* diagnosis. Diagnostic imaging is the mainstay for diagnosis of acute thoracolumbar myelopathies in dogs, complimented by ancillary tests such as blood work (hematology, biochemisty, c-reactive protein (CRP)), cerebrospinal fluid (CSF) analysis, and serology and polymerase chain reaction tests for infectious agents. Magnetic resonance imaging (MRI) is the gold standard for the diagnosis of most thoracolumbar myelopathies in dogs.

Imaging characteristics of various specific disease entities, including IVDD subtypes, infectious causes (e.g., discospondylitis), and vascular causes (e.g., FCE) have been described (9–13). Additionally, numerous reports include individual descriptions of findings on imaging studies for specific disorders, including infectious and non-infectious meningomyelitis. Combining transverse, dorsal, and sagittal planes of different types of sequences, differentiation between disorders preferentially affecting certain locations (e.g., focal/multifocal, grey/white matter/both, unilateral/bilateral) in the spinal cord may be achieved to a certain degree. Histopathological examination remains invaluable for more accurate description and certainty, however.

This case report details the clinical, diagnostic imaging, and histopathological findings in a small breed dog with an acute, symmetrical, progressive, painful, T3-L3 myelopathy with loss of nociception. The aim is to highlight the value, pitfalls, and importance of each, as well as to document detailed findings of this case to which the true etiology remains unknown.

## Case description

A 2.5-year-old female entire Pomeranian dog was presented to the neurology department of IVC Evidensia Small Animal Hospital (Arnhem, The Netherlands) for acute paraparesis progressing within 2 days to paraplegia. The referring veterinarian had diagnosed bilateral patellar luxation and had prescribed rest and meloxicam (0.1 mg/kg *per os* q24h) 3 days earlier. Over the weekend, the dog deteriorated quickly and lost the ability to ambulate, urinate and defecate. General examination was unremarkable. Orthopedic examination revealed bilateral patellar luxation. Neurological examination showed paraplegia without nociception, a mass reflex upon testing perineal reflexes and withdrawal reflexes in the pelvic limbs and patellar hyperreflexia. Cutaneous trunci reflexes were absent caudal to the level of the 6th thoracic vertebra. Thoracolumbar (para)spinal hyperesthesia was present. The bladder was full and upon manual expression, urethral sphincter resistance was noted consistent with an upper motor neuron bladder. The rest of the examination, including evaluation of mental status and behavior, cranial nerve function tests and proprioceptive and reflex tests of the thoracic limbs, was unremarkable. A neuroanatomical localization of a T3-L3 myelopathy was concluded. Hematological and biochemical blood tests [including c-reactive protein (CRP; 6.1 mg/L, ref. range 0–10.0 mg/L)] were unremarkable.

The top differential diagnosis considered at this point was IVDD (IVDE, possibly with secondary myelomalacia). Other differential diagnoses that were considered at this point included unwitnessed trauma, vascular (hematomyelia), inflammatory (meningomyelitis of unknown origin, suspected immune-mediated), infectious (e.g., protozoal myelitis, canine distemper virus myelitis), anomalies with acute complications, and neoplastic (e.g., lymphoma) etiologies.

**Figure 1:**
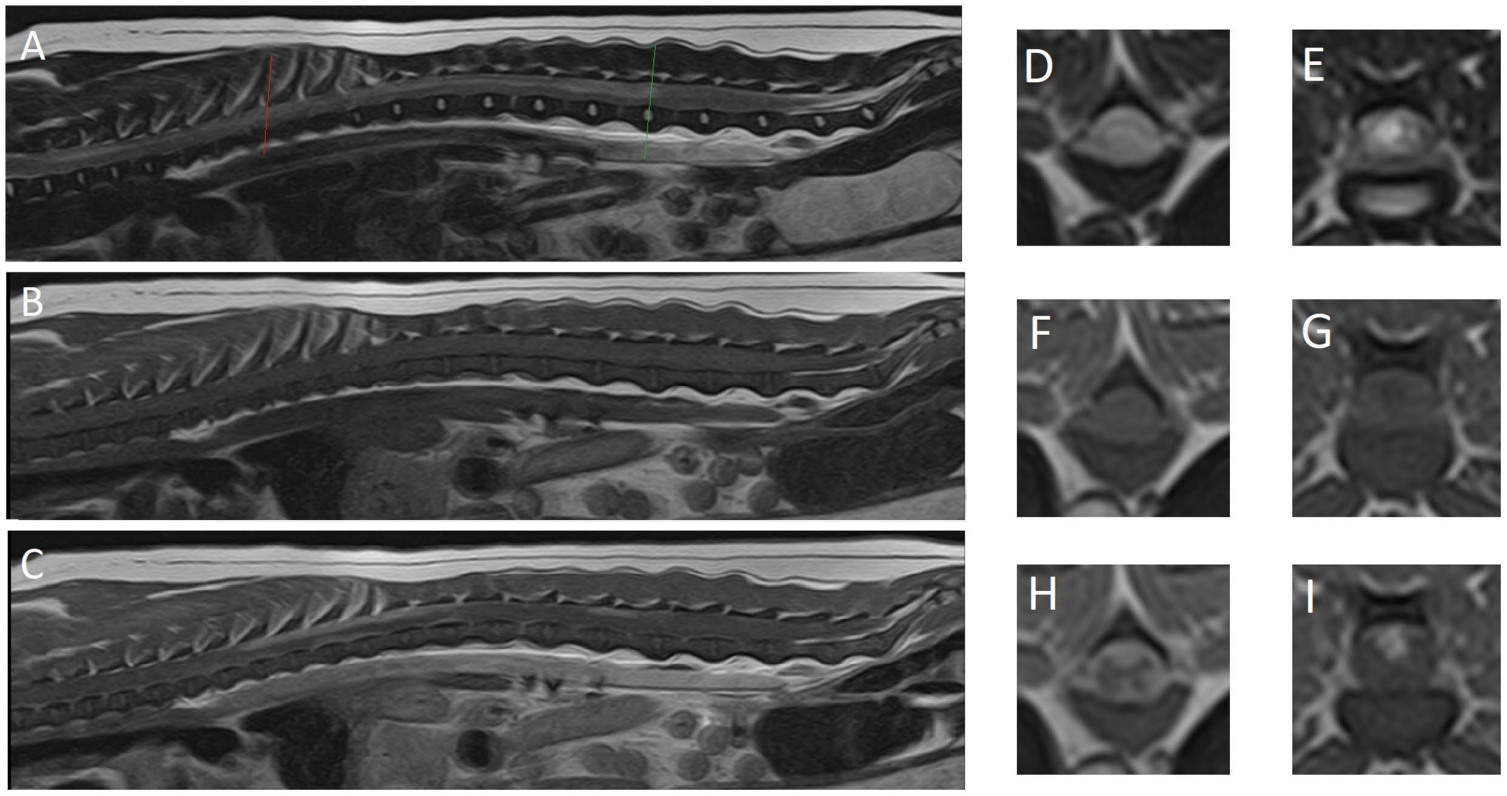
Magnetic resonance images of reported case. (A) Midsagittal plane, (D) transverse plane and (E) transverse plane are T2-weighted images. (B) Midsagittal plane, (F) transverse plane and (G) transverse plane are T1-weighted images. (C) Midsagittal plane, (H) transverse plane and (I) transverse plane are T1-weighted post-contrast images. (D,F,H) The level of T8 (red line). (E,G,I) The level of L3-4 intervertebral disc (green line).

A high-field magnetic resonance imaging (MRI; 1.5 T Canon Vantage Elan) study of the thoracolumbar spinal cord from the level of the 1st thoracic vertebra to the sacrum was performed under general anesthesia. Premedication included 0.4 mg/kg methadone and 6 μg/kg dexmedetomidine, followed by 3 mg/kg propofol in boluses to effect for induction of anesthesia. Isoflurane 2–3% was used for maintenance of anesthetic plane. Performed sequences included: sagittal T2-weighted (T2W), sagittal short tau inversion recovery (STIR), sagittal T1W, dorsal STIR, transverse T2W, transverse T1W, transverse T2*, transverse post-contrast T1W, sagittal post-contrast 3D T1W and transverse post-contrast T1W with fat saturation. The MR images revealed no sign of intervertebral disc disease, nucleus pulposus signal of all imaged intervertebral discs being T2 hyperintense. There was a patchy ill-defined intramedullary lesion of variable extent, but seen from T1 to L4 (Figure 1). Both gray and white matter were affected. The entire cross-sectional area of the cord was affected at the level of T9. The lesion was heterogeneous, moderate to marked T2W hyperintense when compared to the rest of the cord and T1W isointense. The spinal cord parenchyma showed moderate, patchy, contrast enhancement from T7 to T12 and L1 to L3. The cord appeared swollen at those levels and the surrounding meninges were thickened and contrast enhancing, including enhancement along the ventral medial fissure at the level of T9. A lumbar CSF tap was unsuccessful.

The main differential diagnoses that were considered at this point included inflammatory (meningomyelitis of unknown origin, suspected immune-mediated), infectious (e.g., protozoal myelitis, canine distemper virus myelitis) and neoplastic (e.g., lymphoma) etiologies.

Medical treatment was initiated with dexamethasone (0.4 mg/kg once IV), prednisolone (1 mg/kg q12h *per os*), and methadone based on pain scores (0.2–0.5 mg/kg q4-8h). An indwelling urinary catheter was placed and intravenous fluid therapy initiated (Ringer’s lactate solution 3–5 mL/kg/h). Prognosis was grave and after 3 days without return of nociception, the dog was euthanized (by intravenous administration of 140 mg/kg pentobarbital) according to the owners’ wishes.

Post-mortem gross and histopathological examination of the brain and spinal cord was performed. Macroscopically, the thoracolumbar segments of the spinal cord were swollen and showed yellowish discoloration through intact dura mater. After fixation in 10% buffered formalin and sectioning of the affected thoracolumbar segments, malacic areas were identified in the dorsal and ventral funiculi.

**Figure 2:**
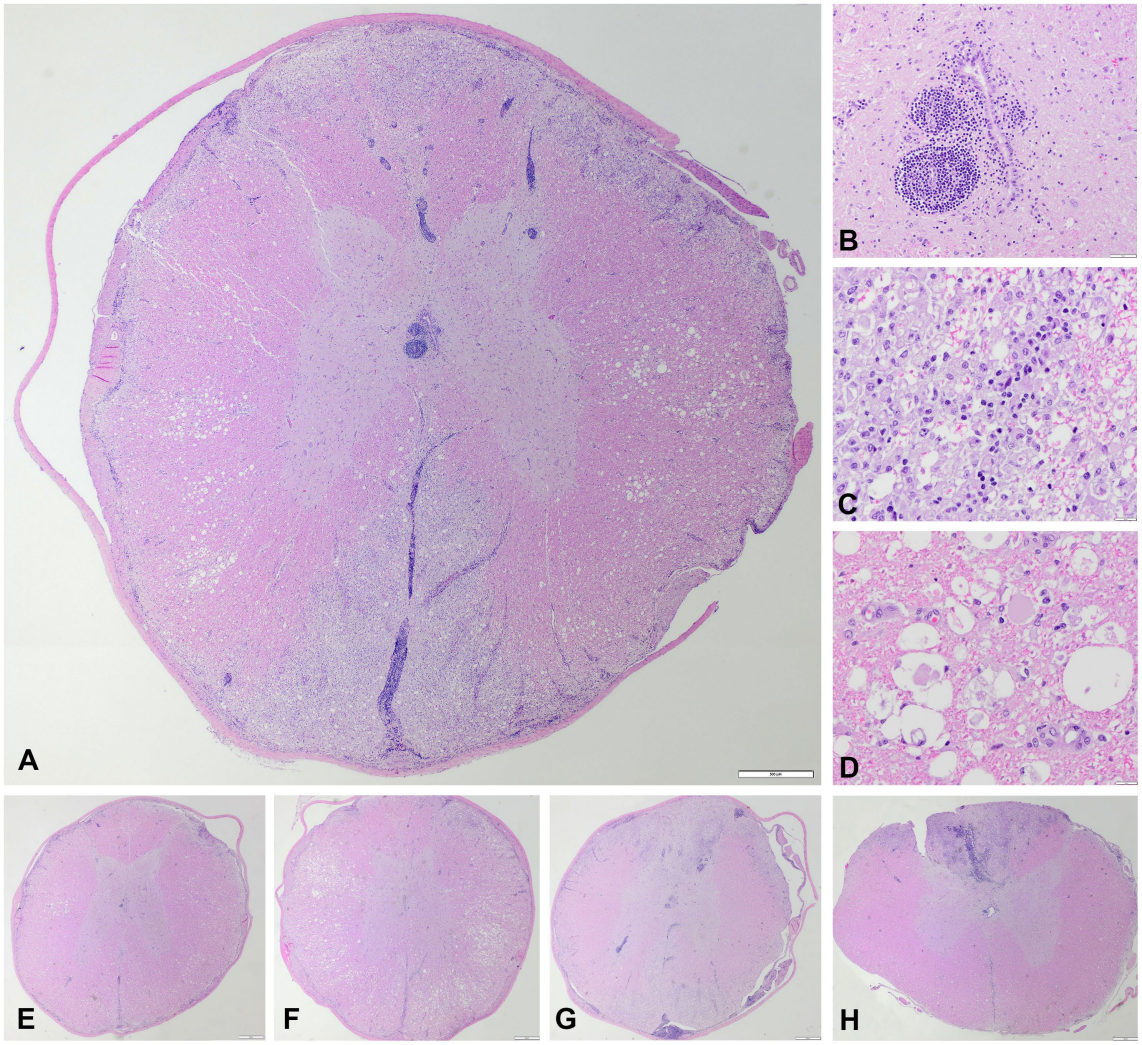
Microscopic images, hematoxylin and eosin. **(A)** Transversal section of the thoracic spinal cord at the level of T9 with bilateral fairly symmetrical necrotizing and lymphohistiocytic leukomyelitis of the dorsal, lateral, and ventral funiculi with a vascular pattern affecting the ventral spinal artery and dorsolateral arteries, and their main branches (vertical and sulcal), degenerative changes of the myelin sheaths and axons in the lateral funiculi, and non-necrotic gray matter with lymphocytic perivascular cuffing and multifocal lymphohistiocytic infiltration within the leptomeninges. Scale bar = 500 μm. **(B)** Lymphocytic perivascular cuffing in the grey matter adjacent to the central canal. Scale bar = 50 μm. **(C)** Detail of the dorsal and lateral funiculi, necrotizing and lymphohistiocytic leukomyelitis with presence of many gitter cells. Scale bar = 20 μm. **(D)** Detail of the lateral funiculi, myelin sheaths vacuolation with marked swelling of axons (spheroids). Scale bar = 20 μm. **(E)** Transversal section of the thoracic spinal cord at the level of T3. Scale bar = 500 μm. **(F)** Transversal section of the thoracic spinal cord at the level of T6. Scale bar = 500 μm. **(G)** Transversal section of the thoracic spinal cord at the level of T12. Scale bar = 500 μm. **(H)** Transversal section of the thoracic spinal cord at the level of L3/4. Scale bar = 500 μm. The craniocaudal extent of the lesions, with preferential involvement of the dorsal funiculi at the lumbar segment in **H**, is appreciated in **E–H**.

Microscopic examination of five hematoxylin and eosin-stained T3-L3 spinal cord sections confirmed an inflammatory process mostly restricted to spinal cord white matter and leptomeninges (Figure 2). This process was characterized by a bilateral and fairly symmetrical, severe necrotizing leukoencephalitis (Figure 2A) with perivascular lymphocytic cuffs (Figure 2B). At the epicenter of the lesions, at segments T9-T12, both dorsal and ventromedial funiculi had marked pallor and were hypercellular. Large numbers of histiocytes infiltrated, compressed, and effaced nerve fibers at those locations totally effacing the spinal white matter architecture. Numerous axons were enlarged (spheroids), and there were empty myelin sheaths, and myelin breakdown accompanied by large macrophages, gitter cells found within dilated myelin sheaths that contain axonal debris, all admixed with a few lymphocytes and neutrophils (Figures 2C,D). Similar granulomatous inflammatory lesions also affected the most submeningeal and superficial white matter, circumferentially, which appear pale, edematous, and hypercellular. No such lesions were found in the gray matter of the spinal cord. At T9-T12, visualization of the meninges confirmed large lymphocytic infiltrates surrounding both the ventral and the dorsal spinal arteries as well as their vascular tree along the leptomeninges (Figures 2A,G,H). The central branch at the ventral median fissure was populated by a dense population of lymphocytes with a few monocytes and neutrophils that upon entry into the gray matter expanded a few Virchow-Robin spaces surrounding the central canal of the ventral horns. Similarly, branches of the dorsal spinal arteries resulted in perivascular lymphocytic cuffs and scattered lymphocytes in the neuropil of the dorsal horns. Neuronal necrosis was not observed in any section. There was no evidence of vasculitis, thrombosis, vascular necrosis, or thromboembolisms in any of the examined sections.

Cranial to but also at T9 (Figures 2A,E,F), spinal cord lateral funiculi multifocally showed evidence of axonal tract degeneration in the form of axonal spheroids and digestion chambers with reactive astrocytes and capillaries (Figures 2E,F). The inflammatory process in those rostral spinal cord sections was minimal and restricted to a few immune cells infiltrating some perivascular spaces.

Ancillary special stains to aid the detection of infectious agents, included a PAS (periodic acid-schiff), Grocott’s methenamine silver stain and a Giemsa stain, and yielded a negative result. Immunohistochemistry for the detection of canine distemper virus, Toxoplasma gondii, *Neospora caninum* and canine parvovirus also provided negative results. Real-time polymerase chain reaction (PCR) tests on spinal cord tissue for *Bartonella* spp., canine distemper virus, *Cryptococcus neoformans*, *Neospora* spp., and *Toxoplasma gondii* were negative as well.

The final morphological diagnosis was a severe, segmental, bilateral and fairly symmetrical, necrotizing lymphohistiocytic leukomyelitis, with a non-suppurative angiocentric leptomeningitis. A specific etiology was not identified.

## Discussion

Despite not being able to reach a specific etiological diagnosis in this case, we considered this case important to document for a number of reasons that we will discuss in the following paragraphs.

First, the value and pitfalls of using clinical reasoning to determine the most likely differential diagnosis are highlighted by the change in number and types of differential diagnoses considered in this case. The top differential considered after history taking and clinical examination was IVDD (IVDE). This would usually be a surgically treatable disorder. The only consistently prognostically valuable clinical parameter in dogs with thoracolumbar IVDE is the presence or absence of nociception; dogs without nociception in the pelvic limbs or tail have a worse prognosis for recovery (4). In one study (1), loss of nociception in canine spinal cord disease was noted in only 5–6% of cases, with listed causes including IVDE, ischemic myelopathy and acute non-compressive nucleus pulposus extrusion (ANNPE) (1). The grade of neurological disability, mainly the loss of nociception, has been identified as a risk factor for the development of ascending-descending myelomalacia. One large study determined that 14.5% of dogs without nociception developed this disastrous complication, while a smaller study found that 21.5% of dogs without nociception died as a consequence thereof despite routine surgery (6, 8). Extensive hemilaminectomy and durotomy were recently proposed and shown to decrease the rate of this complication in dogs (8, 14, 15). In our case, nociception was absent which prompted swift diagnostic imaging to accurately assess the if this dog could have benefitted from surgery. The diagnostic imaging results resulted in a much different plan. Therefore, whilst clinical reasoning is of great value to clinicians, uncommon or less expected diagnosis should not be discounted early on. This is most important when owner are informed ahead of diagnostic test results on the possible outcomes of either the tests themselves or, prematurely, of recommended procedures that follow on the results of those tests.

Second, the value and limitations of MRI in diagnosing or excluding differentials from the differential diagnosis list is reflected in this case report. MRI findings, while very suggestive, were not confirmatory of an inflammatory process. No IVDD was identified on the images and subsequently excluded from the differential diagnosis list. Since the CSF tap failed, no further clinical indications were available to corroborate the suspicion of inflammation or possibly identify neoplastic cells. The post-mortem histopathology confirmed the suspicions of inflammation, but also provided us with the opportunity to assess the similarities between the diagnostic imaging findings (Figure 1) with the histopathological findings (Figure 2). We would like for the reader to compare these alongside each other and appreciate the way the MRI findings largely reflect the histological findings, but also appreciate that the histopathological findings are even more extensive than can be seen on the MR images.

Third, the value and limitations of histopathological examination are highlighted by this case. The changes in the spinal cord parenchyma and meninges was detailed and confirmed that the pathological process was indeed severe as suspected based on the clinical characteristics and imaging findings, with necrosis of the affected tissue. Despite efforts to identify a specific etiology, none was found. Therefore, one may conclude that the term ‘meningomyelitis of unknown origin’ and more specifically since histologically characterizeable ‘necrotizing leukomyelitis and meningitis of unknown origin’. The suffix ‘-of unknown origin’ is often interpreted as ‘likely immune-mediated’. This may be the case, but maybe not. As said in the introduction, a number of etiological categories may be characterized by primary or secondary necrotizing and inflammatory histopathological findings. We deliberately included in the descriptions the term ‘fairly’ for annotation to the “symmetrical” nature of the lesions. The most severely affected segments of the spinal cord were included for examination (segments T8–T10). At the level of these segments, the lesions were severe and fairly symmetrical. However, this is most likely not reflective of the nature of the underlying etiology (i.e., inflammatory process), but rather reflects the effect of the passing of time in an initially multifocal disease process. The authors suspect that the lesions were likely multifocal in nature at the start and that the passing of time led to confluence of the lesions. This, in combination with the restricted anatomy of the spinal cord (transversely at least), can account for the end-stage lesion aspects seen and described in this report. After deliberation with other veterinary (neuro)pathologists, concluded that the necrotizing changes are most likely secondary to a combination of the inflammatory processes as well as ischemia linked to pressure effects of the inflammation on the blood vessels of the spinal cord parenchyma. The distribution of the lesions in the regions of the larger blood vessels and the bilaterally fairly symmetrical pattern seemed to us suggestive thereof. Also, the extent of changes and damage in the grey matter were clearly surpassed by those in white matter. Necrotizing meningomyelitis may arguably be applicable to not limit the morphological diagnosis to the spinal cord white matter and include the abnormalities in grey matter. Still, the authors concluded that the severity of the lesions in white matter preferentially over grey matter merits the inclusion of the prefix leuko- in this case. Finally, although we performed a number of ancillary tests including IHC and PCR, we cannot exclude infectious causes of the identified pathological changes due to lack of testing for other agents as well as imperfect sensitivity and specificity of these tests. Recent reports on tick-born encephalitis in dogs revealed mostly bilateral and symmetrical brain changes with MRI (16, 17). Other etiologies more commonly linked to bilateral and symmetrical changes would include metabolic, toxic, nutritional, and degenerative etiologies, but none of these categories are associated with the changes identified in this case to the knowledge of the authors. Due to lack of identification of an infectious cause, we preferentially consider an auto-immune basis to the changes seen in this case, although we acknowledge the fact that we cannot be entirely certain. In this light, the term ‘necrotizing leukomyelitis and myelitis of unknown origin’ could be applied.

Meningomyelitis in dogs can be caused by an infection, but is suspected to occur alternatively also as suspectedly immune-mediated disease (18–21). Several studies looking for infectious causes of meningoencephalomyelitis or meningoencephalitis have not identified infectious agents, leading to the continued use of the suffix ‘-of unknown origin’ or similar suffices (22, 23). Similar studies focusing on MUO of the spinal cord only have not been performed. Necrotizing meningomyelitis of unknown origin or necrotizing forms of meningoencephalitis of unknown origin (MUO) affecting only the spinal cord are reported rarely in dogs of various breeds, both small (including toy) and large (e.g., hound) (18, 19) and mostly young to middle aged dogs (18–20). Neurological signs are diverse, but general proprioceptive ataxia, paresis, and paraspinal hyperesthesia are the most commonly reported clinical signs (18, 19). The case reported here is unusual with regard to the severity of the clinical presentation, being presented with paraplegia without nociception and both urinary and fecal incontinence. In one study documenting MUO affecting the spinal cord only, not a single case was documented to be (tetra- or) paraplegic without nociception (18). Most cases had involvement of the cervical spinal cord segments. Urinary- and/or fecal incontinence is reported in a minority (10%) (18). In that study, a single case of a 3-year-old female neutered Yorkshire terrier presented with ambulatory tetraparesis was diagnosed with necrotizing meningoencephalomyelitis via histopathological examination (18). Additionally, a 2-year-old female neutered Weimaraner with acute necrohemorrhagic myelitis and vasculitis, that was presented at a veterinary clinic with ambulatory paraparesis progressing to non-ambulatory status, was published as case report (19). As for consistency of the underlying histopathological findings, signs of vasculitis and hemorrhage as reported in the Weimaraner case were not found in our case (19) and no detailed histopathological findings were given in the study on the Yorkshire terrier (18).

Specifically in regards to the case reported here, the authors would like to draw the readers’ attention to some important considerations.

In our case, a lumbar CSF tap was unsuccessful and CSF analysis could not be performed. The added value of CSF analysis in cases of inflammatory myelopathies has been demonstrated (18, 19, 21, 24, 25). One study found a pleocytosis in CSF in 100% of cases of ‘spinal-only’ MUO. Total protein measurement was above reference values in 17/18 dogs (94%). In contrast, a leukocytosis in complete blood counts was only present in 2 dogs (10%) while lymphopenia was present in six dogs (29%). Lumbar CSF taps are reported to be unsuccessful in 13.9% of cases where it was attempted (26). In retrospect, cisternal CSF tap, though likely yielding a less representative sample for analysis in case of myelopathies (27, 28), might have been considered for the case reported here. General bloodwork including a complete blood count revealed no abnormalities and serum CRP was within reference range in our case. This finding is consistent with CRP being normal in most cases of MUO as well (29). For MUO (not for spinal-only MUO or non-infectious meningomyelitis), numerous studies have focused on findings specific and reliable biomarkers (29). As this search continues, future studies could also focus on their value in the diagnosis of meningomyelitis of unknown origin in dogs.

MRI findings in our case consisted an extensive area of (likely confluent) multifocal T2W hyperintense lesions that were T1W isointense and showed parenchymal (spinal cord) as well as meningeal contrast enhancement. MRI features of meningomyelitis of unknown origin in dogs have been reported (18). These include: focal (71%), multifocal (19%) or no lesions (10%); ill-defined, intramedullary, hyperintense lesions on T2W images that are isointense on T1W images; parenchymal contrast enhancement (86%); and meningeal contrast enhancement (81%). Meningeal contrast enhancement could be the only finding (18). Concurrent brain lesions may be identified on MRI, which would suggest meningoencephalomyelitis that can be confirmed by histological examination (18). The extensive parenchymal and meningeal contrast enhancement seen on post-contrast studies as performed in our case provide valuable information to give preference to a diagnosis of meningomyelitis.

In the case reported here, treatment was limited to immunosuppressive treatment with dexamethasone and prednisolone. In a study including 21 dogs with “spinal-only MUO,” medical treatment and the results thereof were reported (18). All dogs were treated with immunosuppressive doses of corticosteroids (dexamethasone 0.3–0.5 mg/kg/day or prednisolone 2–4 mg/kg/day). Fourteen dogs (67%) received additional treatment with cytosine arabinoside in various protocols. Three dogs (14%) showed deterioration despite treatment. There is no definitive proof that additional treatment (e.g., with cytosine arabinoside) to corticosteroids provides benefit in cases of MUO (30, 31). Future studies should provide more information on the possible benefits thereof (32).

Euthanasia was elected for the dog in this case report due to a grave prognosis based on the severity of initial clinical signs including absent nociception and incontinence, and no sign of improvement after initiation of treatment. Based on two larger studies of dogs with meningomyelitis (one including only a non-infectious etiology, the other including both infectious and non-infectious etiologies), approximately 50% of dogs with this diagnosis die (either by euthanasia or naturally) despite attempts at treatment (18, 19).

In the scope of comparative medicine or One Health, in human medical literature the term “(idiopathic) transverse myelitis” is used to describe an acute, bilateral inflammation of the spinal cord with either infectious or non-infectious (idiopathic) causes (33–39). Many patients in the most severely affected category will exhibit loss of sensation and incontinence. MRI findings largely overlap those reported for “spinal-only” MUO in dogs (18, 35, 36). The percentage of cross-sectional area of the spinal cord that is affected varies, but often exceeds two thirds (35). In our case, 100% of the cross-sectional area was affected at the level of the T9 vertebra. This feature does not necessarily correlate to clinical severity in humans (33, 35, 36). Specific MRI features can also be of value in determining the likelihood of idiopathic transverse myelitis versus infectious causes of transverse myelitis (36). Future studies may provide more insights on the value of MRI for the differentiation of infectious versus non-infectious (suspected immune mediated) causes of meningomyelitis. Still, it is clear also from human medical literature that *in vivo* diagnoses of (idiopathic) transverse myelitis cannot be regarded as definitive diagnoses (40); histopathological diagnoses may differ in a large number of suspected cases. In our case, the diagnosis of severe, multifocal, bilateral and fairly symmetrical, multifocal, lymphohistiocytic necrotizing leukomyelitis and meningitis with a vascular pattern affecting the ventral spinal artery and dorsolateral arteries, and their main branches (vertical and sulcal) was based on post-mortem histopathology of the entire central nervous system. This particular pattern of inflammatory lesions in the spinal cord has not been documented before in dogs to the knowledge of the authors.

In conclusion, this case report underlines the importance of including meningomyelitis in the differential diagnosis list of dogs presented for acute progressive neurological signs referable to a myelopathy. Clinical reasoning, diagnostic tests including diagnostic imaging, and histopathological examinations all have their own value and pitfalls or limitations to consider.

## Data availability statement

The original contributions presented in the study are included in the article/supplementary material, further inquiries can be directed to the corresponding author.

## Ethics statement

Ethical approval was not required for the studies involving animals in accordance with the local legislation and institutional requirements because the animal was treated in accordance with the local legislation and institutional requirements. Written informed consent was obtained from the owners for the participation of their animals in this study.

## Author contributions

KS: Conceptualization, Data curation, Investigation, Visualization, Writing – original draft. LG: Investigation, Visualization, Writing – review & editing. EW: Investigation, Visualization, Writing – review & editing.
